# Prevalence and complications of hypouricemia in a general population: A large-scale cross-sectional study in Japan

**DOI:** 10.1371/journal.pone.0176055

**Published:** 2017-04-27

**Authors:** Masanari Kuwabara, Koichiro Niwa, Akira Ohtahara, Toshihiro Hamada, Satoshi Miyazaki, Einosuke Mizuta, Kazuhide Ogino, Ichiro Hisatome

**Affiliations:** 1 Department of Cardiology, Toranomon Hospital, Tokyo, Japan; 2 Division of Renal Diseases and Hypertension, School of Medicine, University of Colorado Denver, Aurora, Colorado, United States of America; 3 Department of Cardiology, Cardiovascular Center, St. Luke's International Hospital, Tokyo, Japan; 4 Department of Cardiology, Sanin Rosai Hospital, Yonago, Tottori, Japan; 5 Department of Community-Based Family Medicine, Tottori University Faculty of Medicine, Yonago, Tottori, Japan; 6 Department of Cardiovascular Medicine, Masao Fujii Memorial Hospital, Kurayoshi, Tottori, Japan; 7 Department of Clinical Laboratory, Tottori University Hospital, Yonago, Tottori, Japan; 8 Division of Regenerative Medicine and Therapeutics, Institute of Regenerative Medicine and Biofunction, Tottori University Graduate School of Medical Sciences, Yonago, Tottori, Japan; University of Colorado Denver School of Medicine, UNITED STATES

## Abstract

**Backgrounds:**

Hypouricemia was reported as a risk factor for exercise-induced acute renal injury (EIAKI) and urinary stones. However, the prevalence of kidney diseases among hypouricemic subjects has not been evaluated. This study was conducted to clarify the prevalence of hypouricemia and the association of hypouricemia with kidney diseases by using a large-scale Japanese population data.

**Methods:**

This study is a retrospective cross-sectional study at the Center for Preventive Medicine, St. Luke’s International Hospital, Tokyo, Japan, and Sanin Rousai Hospital, Yonago, Japan. We analyzed the medical records of 90,143 Japanese subjects at the center in St. Luke’s International Hospital, Tokyo, and 4,837 subjects in Sanin Rousai Hospital, Yonago, who underwent annual regular health check-up between January 2004 and June 2010. We defined hypouricemia as serum uric acid level of ≤2.0 mg/dL. We checked the medical history of all the study subjects and compared the rates of complications including urinary stones and kidney diseases among those with or without hypouricemia.

**Results:**

The prevalence of hypouricemia was 0.19% in St. Luke’s International Hospital, Tokyo, and 0.58% in Sanin Rousai Hospital, Yonago. The prevalence of hypouricemia in women was larger than that in men both in Tokyo (0.31% vs 0.068%, p<0.001) and in Yonago (1.237% vs 0.318%, p<0.001). Among 172 hypouricemic subjects (30 men), the rates of previous urinary stones and kidney diseases (including nephritis/nephrosis) were 1.2% (3.3% men, 0.7% women) and 2.3% (10% men, 0.7% women), respectively. Hypouricemic men had a 9-fold higher rate of previously having kidney diseases compared to non-hypouricemic men (p<0.001). However, the rates of other diseases including urinary stones were not significantly different between the two groups.

**Conclusions:**

Hypouricemia was associated with a history of kidney disease especially in men.

## Introduction

Hypouricemia was first reported in 1950, which is mainly caused by impaired renal transport of uric acid.[1] Hypouricemia is caused by a consequence of not only increased renal clearance of uric acid but also decreased formation of uric acid.[2] There are some specific diseases that cause hypouricemia. Fanconi syndrome has a functional defect of proximal tubules, causing hypouricemia,[3] while xanthinuria and the deficiency of purine nucleotide phosphorylase are also some of the causes of hypouricemia, but these are very rare.[4, 5] Wakasugi et al. reported the prevalence of hypouricemia in Japan was 0.21% in men and 0.39% in women.[6] Son et al. reported the prevalence of hypouricemia in Korea was 0.53% among outpatients and 4.14% among inpatients (1.39% overall).[7] However, there was little study that compared the prevalence of hypouricemia between different geographical areas in the same periods.

It is known that hypouricemia is often accompanied with urinary stones and exercise-induced acute kidney injury (EIAKI) especially in hereditary hypouricemic subjects.[8, 9] Since uric acid acts as an anti-oxidant to protect endothelial function, hypouricemia causes EIAKI through spasm of renal artery characterized by nausea/vomiting, loin pain, abdominal pain and general fatigue. On the other hand, excess urinary excretion of uric acid frequently leads to the uric acid crystal formation, causing the urinary stones in hypouricemic subjects. Ichida et al. reported the prevalence of urinary stones (8.5%) and EIAKI (6.5%) in hereditary renal hypouricemic subjects based on genetic testing, but the study population was very small (only 71 subjects) and had some bias because all the subjects were regularly evaluated in a university hospital.[10] The number of study subjects was much smaller than results derived from a large database, providing hypouricemia non-specifically. We have to conduct further study about hypouricemia in a larger population.

Renal hypouricemia (RHUC) is mainly caused by urate transporter mutations such as SLC22A12 (URAT1): RHUC type 1 and SLC2A9 (GLUT9/URATv1): RHUC type 2,[11–15] because urate transporter works to reabsorb uric acid mainly from proximal tubular in kidney. Since hypouricemia is the heterologous disease composed of RHUC, xanthinuria and secondary hypouricemia, it is important to know the prevalence and cause of hypouricemia in a general population. There is also little evidence on how often urinary stones and EIAKI were accompanied with hypouricemia in a general population. We conducted this study to examine the regional differences in prevalence of hypouricemia in Japan and compare the prevalence of urinary stones and kidney diseases between individuals with and without hypouricemia.

## Materials and methods

### Study design and study subjects

This study was a large-scale, cross-sectional study to clarify whether hypouricemia became a risk for diseases such as urinary stones and EIAKI or not in a general population. We used the database on subjects who underwent annual regular health check-up at the Center for Preventive Medicine, St. Luke’s International Hospital, Tokyo, and Sanin Rousai Hospital, Yonago, Japan. We analyzed the medical records of study subjects mentioned above between January 2004 and June 2010. When the study subjects had examinations more than once during the period, we adopted only the first results to avoid double counts. Every subject had almost same physical and laboratory examinations. Some studies were published by using the database in St. Luke’s International Hospital but this study design was completely different from the previous studies.[16–18]

First, we calculated the prevalence of hypouricemia in each sex. We compared the prevalence of hypouricemia between the Pacific side (St. Luke’s International Hospital, Tokyo) and the Japan sea side (Sanin Rousai Hospital, Yonago, 4,837 subjects), since there were no report to study the regional differences of prevalence of hypouricemia in Japan.

Second, we compared general status, vital sign, blood test, the prevalence of current diseases, such as hypertension, diabetes mellitus, dyslipidemia, and chronic kidney diseases (CKD), and the rates of having current or previous medical conditions, such as urinary stones, kidney diseases including nephritis/nephrosis, asthma, and depression, between the subjects with and without hypouricemia by using only the database in St. Luke’s International Hospital.

### Definition of hypouricemia, CKD, hypertension, diabetes mellitus and dyslipidemia

Hypouricemia was defined as 2.0 mg/dL and less in serum uric acid level as in some previous studies.[6, 19] We evaluated serum uric acid level only once, and the hypouricemia identified were not verified if they were transient or persistent. This study group might include some transient hypouricemia.[20] CKD was defined as less than 60 ml/min/1.73m^2^ in eGFR, which was calculated using the Japanese GFR equation; eGFR (ml/min/1.73m^2^) = 194 × Cr^-1.094^ × age^-0.287^ (×0.739 if female).[21] Hypertension was defined as systolic blood pressure (BP) of ≥140 mmHg and/or diastolic BP of ≥90 mmHg according to the Japanese Society of Hypertension guidelines (JSH 2014).[22] This study also included the subjects who had current anti-hypertension medication. Two BP examinations were taken, and the mean systolic and diastolic BP of each of the participants were calculated from the recorded measurements. Diabetes mellitus was defined as an HbA1c (National Glycohemoglobin Standardization Program) level of 6.5% and more according to International Expert Committee.[23] This study also included the subjects who had current medication for diabetes mellitus. Dyslipidemia was defined as low-density lipoprotein cholesterol level ≥140 mg/dl, high-density lipoprotein cholesterol level <40 mg/dL and/or triglyceride level ≥150 mg/dL according to Japan Atherosclerosis Society guidelines.[24] This study also included the subjects who had current medication for dyslipidemia.

### Statistical analysis

Statistical analyses were performed using SPSS Statistics software (IBM SPSS Statistics version 22 for Windows; IBM, New York). Background data were expressed as mean (standard deviations) or as percent frequency, as appropriate. Bivariate associations between demographic and clinical characteristics were compared between subjects with and without hypouricemia (serum uric acid level of ≤2.0 mg/dL) by using t-test for distributed variables and χ^2^ test for categorical data. Statistical significance was set at α = 0.05. All statistical analyses were two-sided. All relevant data are within the manuscript and its Supporting Information files (S1 Data).

### Ethics statement

We adhere to the principles of the Declaration of Helsinki. All data were collected and compiled in a protected computer database. St. Luke’s International Hospital ethics committee approved this study. We had consents from all the subjects by comprehensive agreement method in the hospital. The ethics committee approved this consent procedure. This study is a retrospective cohort study by using existing data, in which individual data were anonymized and there was no personal information identified. So, this study does not have any ethics problems. We have also published some articles by using the database. [16–18, 25]

## Results

### Study subjects characteristics

Of the 90,143 subjects in St. Luke’s International Hospital, five subjects were excluded due to lack of blood test data. Two were less than 18 years old, and two lacked BP data, but we included them because we could evaluate their serum uric acid level and medical history. We enrolled 90,138 (99.99%) subjects in the study. Of the 4,837 subjects in Sanin Rousai Hospital, we were able to enroll all the subjects. The mean age (standard deviations) of the study subjects at St. Luke’s International Hospital and Sanin Rousai Hospital was 46.3 (12.0) years and 50.8 (9.7) years. The rate of men was 49.1% and 71.6%, respectively.

### Regional differences of prevalence of hypouricemia in Japan

**Fig 1:**
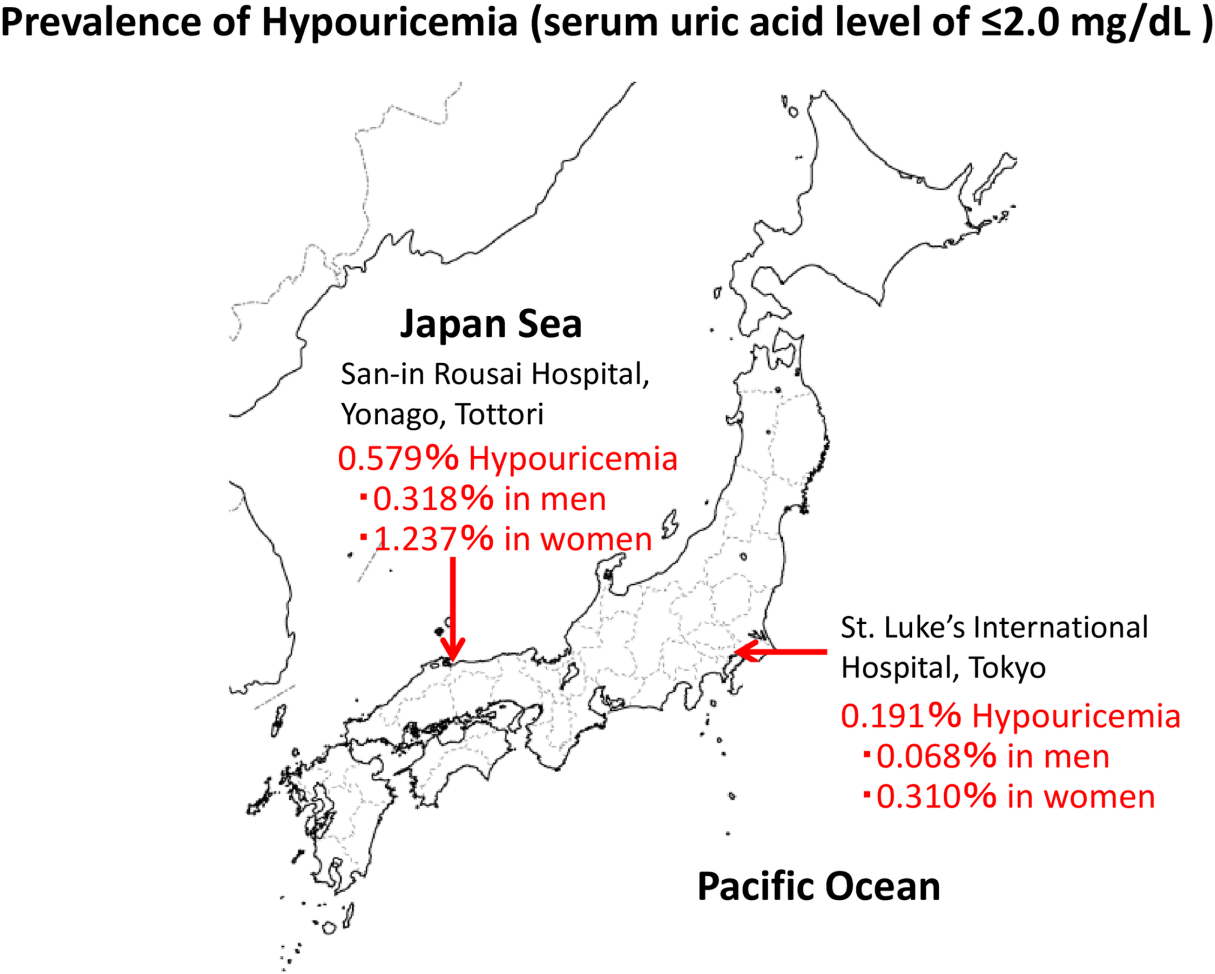
Prevalence of hypouricemia (serum uric acid level of ≤2.0 mg/dL). The prevalence of hypouricemia in Yonago (Japan Sea side) is significantly higher than that in Tokyo (Pacific side) (0.579% vs 0.191%, p<0.001). The prevalence of hypouricemia in women was larger than that in men both in Tokyo (0.310% vs 0.068%, p<0.001) and in Yonago (1.237% vs 0.318%, p<0.001).

First, we calculated the prevalence of hypouricemia in this study’s population. The number (prevalence) of hypouricemia was 172 (0.191%) at St. Luke’s International Hospital, Tokyo (Pacific side) and 28 (0.579%) at Sanin Rousai Hospital, Yonago (Japan Sea side). The prevalence of hypouricemia in Yonago was more than threefold higher than that in Tokyo (p<0.001). The prevalence of hypouricemia in women was fourfold larger than that in men both in Tokyo (0.310% vs 0.068%, p<0.001) and in Yonago (1.237% vs 0.318%, p<0.001) (Fig 1).

### Distribution of hypouricemia among each serum uric acid level and age

**Fig 2:**
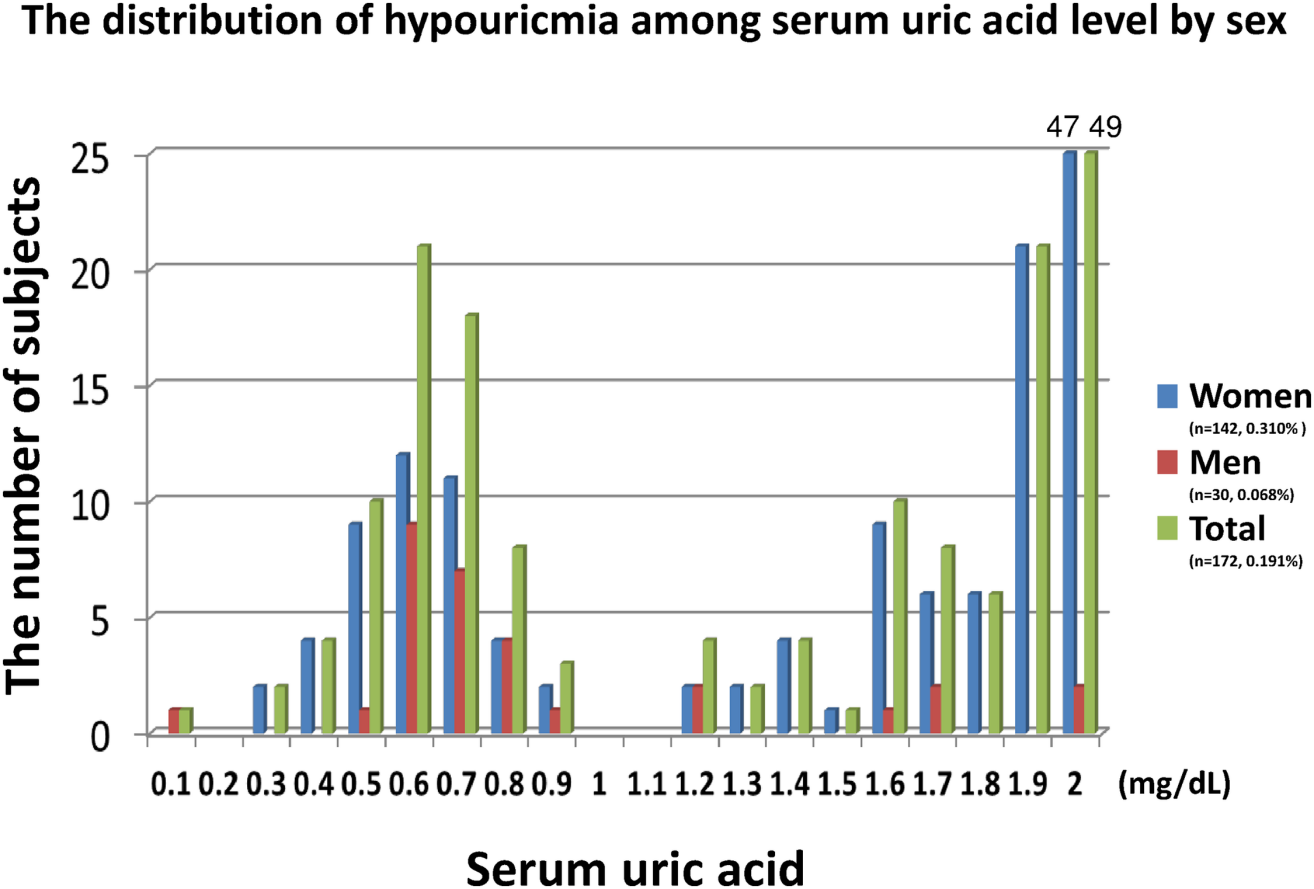
The distribution of hypouricemia among serum uric acid level by sex. The number of the women subjects whose serum uric acid of 2.0 mg/dL was 47. The number of the total (men and women) subjects whose serum uric acid of 2.0 mg/dL was 49. These numbers were above this graph limit (25 subjects), and we showed the numbers in the graph.

**Fig 3:**
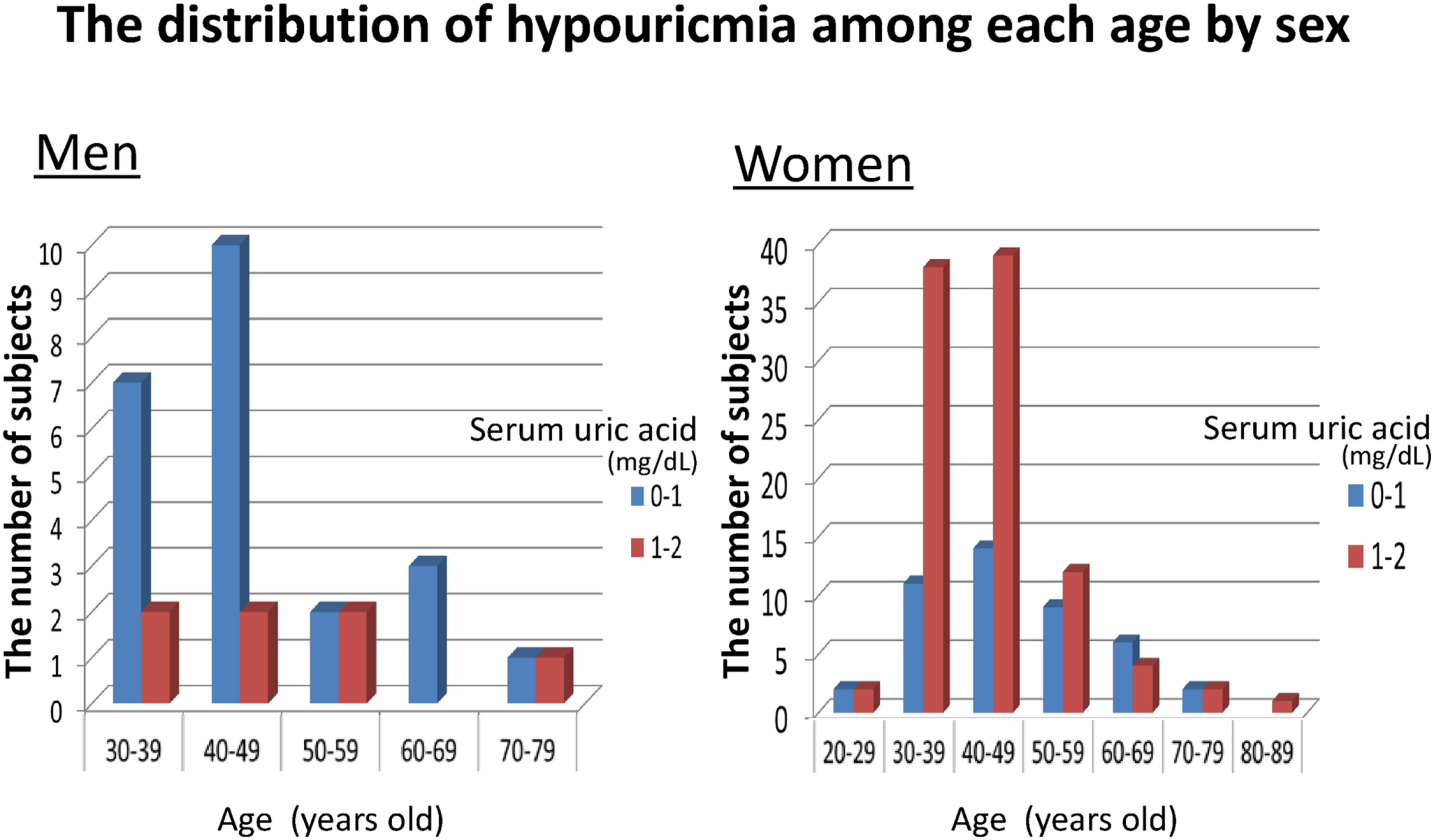
The distribution of hypouricemia among each age by sex. In women, the number of hypouricemia subjects was decreased with age.

Second, we checked the distribution of hypouricemia at St. Luke’s International Hospital among each serum uric acid level (Fig 2) and among each age (Fig 3). There were three peaks of prevalence of hypouricemia along with the serum uric acid level. The first peak of its frequency was at approximately 0.6 mg/dL serum uric acid, the second one was at approximately 1.6 mg/dL serum uric acid, and the third peak was at approximately 2.0 mg/dL serum uric acid. The number of the subjects with serum uric acid level of 2.0 mg/dL was 49 (47 women), which was around one third of the hypouricemic women. When we included the subjects whose serum uric acid level was ≥1.8 mg/dL, the number was 76 subjects (74 women), which was more than one third of all the hypouricemic subjects and more than half of hypouricemic women (Fig 2). The number of hypouricemic women was lower with age, but the number in men did not change with age (Fig 3).

### Characteristics and medical history of hypouricemic subjects

Third, we compared the characteristics and medical history of the study subjects with hypouricemia and without hypouricemia from St. Luke’s International Hospital (Table 1). The age was not significantly different between subjects with and without hypouricemia, but the rate of men with hypouricemia was significantly lower than that of men without hypouricemia (17.4% vs 49.1%, <0.001). We also compared these items by sex (Table 2). The prevalence of CKD between hypouricemic and non-hypouricemic subjects was not significantly different both in men (6.7% vs 6.9%, p = 0.95) and women (3.5% vs 4.5%, p = 0.56), but hypouricemic subjects had better kidney function than non-hypouricemic subjects. Hypouricemic women revealed lower rates of smoking habits and dyslipidemia, and lower levels of serum sodium, calcium, and phosphate, compared to non-hypouricemic women.

**Table 1:** Characteristics of the study subjects.

Total		Hypouricemia	Non-hypouricemia	P
Number of subjects		172	89,966	
Men		17.4%	49.1%	<0.001
Age	years old	44.9 (10.9)	46.3 (12.0)	0.079
Height	Cm	160.7 (7.1)	164.3 (8.7)	<0.001
Weight	Kg	53.9 (11.7)	60.8 (12.4)	<0.001
Body mass index	kg/m^2^	20.7 (3.2)	22.4 (3.3)	<0.001
Smoking		22.1%	40.6%	<0.001
Drinking habits		51.1%	62.0%	0.003
Hypertension		9.3%	15.5%	0.025
Diabetes		2.9%	4.2%	0.40
Dyslipidemia		20.9%	35.6%	<0.001
CKD		4.1%	5.7%	0.35
Serum uric acid	mg/dL	1.36 (0.63)	5.29 (1.41)	<0.001
Creatinine	mg/dL	0.618 (0.145)	0.736 (0.231)	<0.001
eGFR	ml/min/1.73m^2^	104.0 (26.5)	102.3 (26.3)	0.41
Serum sodium	mmol/L	141.0 (2.5)	141.5 (1.8)	0.002
Serum potassium	mmol/L	4.07 (0.29)	4.11 (0.28)	0.11
Serum chloride	mmol/L	106.2 (2.4)	106.1 (1.9)	0.43
Serum calcium	mg/dL	9.02 (0.33)	9.21 (0.33)	<0.001
Serum phosphate	mg/dL	3.41 (0.39)	3.44 (0.43)	0.38
C-reactive protein	mg/dL	0.11 (0.15)	0.13 (0.30)	0.28

Means and standard deviations (SD) are reported using the following format: "mean (SD)"

**Table 2:** Characteristics of the study subjects by sex.

		Men			Women		
		Hypouricemia	Non-hypouricemia	P	Hypouricemia	Non-hypouricemia	p
Number of subjects		30	44,206		142	45,760	
Age	years old	46.9 (11.6)	46.9 (12.1)	0.99	44.4 (10.8)	45.8 (11.9)	0.19
Height	Cm	171.0 (4.3)	170.8 (6.1)	0.81	158.5 (5.4)	158.1 (5.7)	0.37
Weight	Kg	72.8 (11.6)	69.4 (10.2)	0.071	49.9 (6.8)	52.5 (7.8)	<0.001
Body mass index	kg/m^2^	24.9 (3.4)	23.8 (3.1)	0.052	19.9 (2.3)	21.0 (3.0)	<0.001
Smoking		63.3%	61.8%	0.87	13.4%	20.2%	0.042
Drinking habits		80.0%	77.1%	0.70	45.1%	47.5%	0.56
Hypertension		16.7%	20.8%	0.58	7.7%	10.4%	0.29
Diabetes		10.0%	6.4%	0.43	1.4%	2.0%	0.61
Dyslipidemia		46.7%	47.2%	0.95	15.5%	24.3%	0.014
CKD		6.7%	6.9%	0.95	3.5%	4.5%	0.56
Serum uric acid	mg/dL	0.88 (0.47)	6.33 (1.21)	<0.001	1.47 (0.61)	4.40 (0.93)	<0.001
Creatinine	mg/dL	0.795 (0.154)	0.854 (0.234)	0.16	0.581 (0.112)	0.620 (0.160)	0.002
eGFR	ml/min/1.73m^2^	120.7 (25.1)	108.2 (27.3)	0.012	100.4 (25.5)	96.6 (24.0)	0.059
Serum sodium	mmol/L	142.1 (1.63)	141.9 (1.7)	0.47	140.7 (2.6)	141.3 (1.8)	0.013
Serum potassium	mmol/L	4.19 (0.26)	4.17 (0.28)	0.58	4.05 (0.29)	4.05 (0.28)	0.84
Serum chloride	mmol/L	105.9 (2.1)	105.9 (1.9)	0.90	106.2 (2.5)	106.2 (1.8)	0.97
Serum calcium	mg/dL	9.23 (0.40)	9.28 (0.32)	0.39	8.97 (0.30)	9.14 (0.33)	<0.001
Serum phosphate	mg/dL	3.27 (0.36)	3.31 (0.42)	0.62	3.44 (0.39)	3.57 (0.41)	<0.001
C-reactive protein	mg/dL	0.14 (0.19)	0.15 (0.35)	0.91	0.10 (0.13)	0.11 (0.25)	0.46

Means and standard deviations (SD) are reported using the following format: "mean (SD)"

Moreover, we checked the medical history of 172 hypouricemic subjects. There was no subject requiring dialysis, but there were 4 subjects with hepatitis (2 hepatitis B, 2 hepatitis C), and 7 subjects with history of cancer (3 breast cancer, 3 cervical cancer, 2 gastric cancer, 1 both breast and cervical cancer). All the 4 subjects with hepatitis had no medication when they had the medical check-up (1 subject had finished the treatment for hepatitis B). All the 7 subjects with history of cancer had finished the treatment by surgeries and were not taking anti-cancer medications when they had the medical check-up. There was 1 subject with brain tumor (no treatment) but there was no subject with history of encephalopathy. The number of the subjects with history of urinary stones, kidney diseases including nephritis/nephrosis, asthma, and depression both in hypouricemic and non-hypouricemic subjects (Table 3). A history of urinary stones was found in 1.2% hypouricemic subjects (3.3% men, 0.7% women). The rates of previous medical conditions like urinary stones, asthma, and depression were not significantly different between the two groups.

**Table 3:** The differences of medical history between hypouricemia and non-hypouricemia.

	Total			Men			Women		
	HUA	Non-HUA	P	HUA	Non-HUA	p	HUA	Non-HUA	p
Number of subjects	172	89,966		30	44,206		142	45,760	
Urinary stones	2 (1.2%)	3,246 (3.6%)	0.086	1 (3.3%)	2,538 (5.7%)	0.57	1 (0.7%)	708 (1.5%)	0.42
Kidney disease including nephritis/nephrosis	4 (2.3%)	1,118 (1.2%)	0.20	3 (10%)	500 (1.1%)	<0.001	1 (0.7%)	618 (1.4%)	0.50
Asthma	6 (3.5%)	3,224 (3.6%)	0.95	3 (10%)	1,683 (3.8%)	0.077	3 (2.1%)	1,541 (3.4%)	0.41
Depression	2 (1.2%)	1,493 (1.7%)	0.61	0	700 (1.6%)	0.49	2 (1.4%)	793 (1.7%)	0.77

HUA: hypouricemia,

The number of subjects and the rate of each disease are reported using the following format: "number (rate)"

A history of kidney diseases including nephritis/nephrosis was found in 2.3% hypouricemic subjects (10.0% men, 0.7% woman), indicating that hypouricemic men had a 9-fold higher rate of previously having kidney diseases than non-hypouricemic men (10.0% vs 1.1%, p<0.001). Table 4 shows the details of 4 hypouricemic subjects who had a history of kidney diseases. Their kidney function looks completely recovered.

**Table 4:** The details of four hypouricemic subjects who had a history of kidney diseases.

Subject		1	2	3	4
Age	years old	43	62	33	44
Sex		man	man	man	woman
onset of kidney disease	years old	18	36	15	10
Serum uric acid	mg/dL	0.6	0.7	0.6	0.7
Height	Cm	169.9	163.4	170.1	155
Weight	Kg	62.3	83.1	62.8	51.4
Body mass index	kg/m^2^	21.6	31.1	21.7	21.4
Smoking		past	current	no	past
Drinking habits		yes	yes	yes	no
Hypertension		no	yes	no	no
Diabetes mellitus		no	no	no	no
Dyslipidemia		no	yes	no	no
Serum creatinine	mg/dL	0.56	0.63	0.72	0.58
eGFR	ml/min/1.73m^2^	124.3	98.4	101.9	87.8
Proteinuria		negative	negative	negative	negative
urine gravity		1.03	1.03	1.027	1.028
urine pH	ml/min/1.73m^2^	6	5.5	5.5	6
Serum sodium	mmol/L	141	142	141	139
Serum potassium	mmol/L	4.1	4.2	4.5	4.1
Serum chloride	mmol/L	105	106	104	107
Serum calcium	mg/dL	8.9	9.2	9	8.7
Serum phosphate	mg/dL	3.1	3.5	3.5	3.1
C-reactive protein	mg/dL	0.3	1	0.07	0.04

## Discussion

The primary finding of the study was that hypouricemia had a 9-fold higher rate of previously having kidney diseases including nephritis/nephrosis only in men since there were no significant differences of the rate of kidney diseases between hypouricemia and non-hypouricemia in women. In contrast, hypouricemic subjects showed a lower rate of having urinary stones than non-hypouricemic subjects, but it was not significant (1.2% vs 3.6%, p = 0.086). This result was different from previous reports that showed urinary stones was one of the complications of hypouricemia.[10, 26] While it is well-recognized that hereditary renal hypouricemia due to the mutation of urate transporters frequently associated with urinary stones and renal failure, in our study hypouricemia per se did not increase urinary stones both in men and women, indicating the big gap of the rates of complications between hypouricemia per se and hereditary renal hypouricemia. We may also underestimate the rate of previous urinary stones because we could not detect silent urinary stones.

Wakasugi et al. also showed hypouricemia is associated with impaired kidney function.[6] However, our study showed that hypouricemia per se in a general population did not cause severe kidney dysfunction in a long period (Table 2). All the hypouricemic subjects who had a history of kidney diseases had good kidney function in this study (Table 4), which suggested that the kidney diseases associated with these hypouricemic individuals showed a reversible kidney function. Further study is necessary to clarify the relationship between hypouricemia and kidney diseases in a general population.

This study also showed that the prevalence of hypouricemia in Yonago (Japan Sea side) was significantly higher than that in Tokyo (Pacific side) (0.579% vs 0.191%, p<0.001) and was lower than that in Korea (1.39%).[7] While its underlying mechanism remains unknown, there are several possibilities on regional differences of prevalence of hypouricemia in Japan. 1) regional differences of the prevalence of the G774A mutation of SLC22A12 gene responsible for more than 60% of hypouricemic Japanese[27]; 2) different ratios of men to women in the studied population; 3) different circumstances including nutrition between two cities. This study only showed that the prevalence of hypouricemia differs between areas. Further study to clarify the regional differences of hypouricemia is needed.

The prevalence of hypouricemia in this study was smaller than the previous reports described in Japan.[6] Why is the prevalence of hypouricemia in this study lower than the previous studies?[6, 7] In previous studies, the subjects had regular follow-ups in hospitals and the prevalence of hypouricemia in them would be higher than the general population. The subjects of this study were derived from the general population, because they had annual medical check-ups at the center by themselves. A report showed that the prevalence of hypouricemia was 0.18% in the health examination database of the personnel of Japan Maritime Self-Defense Force, Saitama.[12] The subjects of the study were mostly men, and the prevalence of hypouricemia was similar to that in our study. Saitama city is located between Tokyo and Yonago, and the study showed the prevalence of hypouricemia in Saitama was higher than in Tokyo but lower than in Yonago. The result also supported that the prevalence of hypouricemia in Japan Sea side might be higher than that in Pacific side.

The number of hypouricemia in women decreases with age (Fig 3). This result was similar with a previous study.[6] Since estrogen or progesterone decreases the serum uric acid level, the serum uric acid level in women is well-known to significantly increase after menopause,[28, 29] which can explain the reason why the number of hypouricemic women decreases with age. Female hormones were associated with the higher prevalence of hypouricemia in women than that in men, especially in subjects with serum uric acid level of ≥1.8 mg/dL. There were three peaks of prevalence of hypouricemia along with the serum uric acid level (Fig 2), and we consider that the first (0.6mg/dL) and the second peak (1.6 mg/dL) were homozygote and heterozygote genetic urate transporter problems (persistent hypouricemia), and the third peak (2.0 mg/dL) was transient hypouricemia mainly influenced by female hormones because this group subjects were mostly women. Genetic hypouricemia especially in men is strongly associated with EIAKI. On the other hand, transient hypouricemia which is mainly influenced by female hormones is less associated with EIAKI.

There are several limitations in the present study. First, this study was not able to assess hypouricemia as transient or persistent because we checked serum uric acid only once. Some subjects might had some changes in their serum uric acid levels. A study showed that 40% of hypouricemia among outpatients were transient because sometimes hypouricemia is secondary condition, frequently caused either by drug ingestion or systemic diseases.[20] We should consider that the similar rate of hypouricemia might be transient in our study because the prevalence of hypouricemia in women decreases with age (Fig 3) and hypouricemic subjects whose serum uric acid level of ≥1.8 mg/dL were mostly women (74/76), which was more than half of hypouricemic women. In contrast, we could consider that causes of hypouricemia in men were mostly genetic and persistent because hypouricemic men whose serum uric acid level were almost less than 1.8 mg/dL had little estrogen and/or progesterone. Second, this study utilized a retrospective, cross-sectional study design and there is a possibility of selection bias since the information were obtained from subjects of two hospitals only. However, we analyzed almost all the subjects and there was very low deficit rate, thus weakening selection bias. These epidemiological studies always had possible selection bias, but the merit is that there is little laboratory bias because of the same examinations conducted to every subject. Third, we could not get the medical history of EIAKI directly, and we corrected a self-reported history of kidney diseases including nephritis/nephrosis instead. In addition to the previously reported association between hereditary renal hypouricemia and EIAKI, our subjects with hypouricemia were associated with a history of kidney diseases including nephritis/nephrosis. All the hypouricemic subjects who had a history of kidney diseases (Table 4) had completely recovered kidney function, which indicated this kidney diseases were high possibilities of EIAKI because most EIAKI are improved by fluid resuscitation.[30] Fourth, we were not able to detect the cause of hypouricemia because we could not evaluate urine uric acid levels. Most hypouricemia were caused by hyper excretion,[27] but some were caused by low production (xanthine oxidase deficiency). Fifth, we did not analyze the confounding factors between the two populations. Therefore, we just showed the different prevalence of hypouricemia between Yonago and Tokyo. However, confounding factors of hypouricemia except for genetic problems remain unknown. We also showed the prevalence of hypouricemia both in Tokyo and in Yonago by sex because one of the main confounding factors of hypouricemia is sex. Finally, some medicines affect serum uric acid level. Xanthine oxidase inhibitors (e.g., allopurinol, febuxostat, topiroxostat) and uricosuric agents (e.g., probenecid, benzbromarone) are famous medicines for gout and hyperuricemia treatments, but some angiotensin receptor blockers (e.g., losartan, irbesartan), sodium-glucose co-transporter 2 (SGLT2) inhibitors and anti-dyslipidemia drugs (e.g., fenofibrate) facilitate uric acid excretion in the kidney. In contrast, diuretics such as thiazide and furosemide elevate serum uric acid levels.[31] While we checked the medications of all hypouricemic subjects, the specific name of the medications they were taking were not available. However, the number of hypouricemic subjects who were on medications was low. Of 172 hypouricemic subjects, 4 subjects had medication for dyslipidemia, 3 subjects had anti-hypertensive medication, 1 subject had medication for diabetes mellitus, and 1 subject had medication for urinary stones. There was no subject with xanthine oxidase inhibitors or uricosuric agents. After we repeated an analysis in which we excluded these subjects, the results were almost same.

## Conclusions

Hypouricemia was associated with a history of kidney disease especially in men. Hypouricemic men had a 9-fold higher rate of previously having kidney diseases.

## Supporting information

S1 Data (XLSX)
